# Impact of primary cancer history and molecular landscape in therapy-related myeloid neoplasms

**DOI:** 10.3389/fonc.2025.1563990

**Published:** 2025-04-24

**Authors:** Alessandro Costa, Federica Pilo, Martina Pettinau, Eugenia Piras, Clara Targhetta, Rodrigo Rojas, Paola Deias, Olga Mulas, Giovanni Caocci

**Affiliations:** ^1^ Hematology, Department of Medical Sciences and Public Health, University of Cagliari, Cagliari, Italy; ^2^ Hematology and HSCT Unit, “A. Businco” Hospital, ARNAS, Brotzu, Cagliari, Italy

**Keywords:** therapy-related myeloid neoplasm, molecular profiling, latency, TP53 mutation, solid cancer, hematologic cancer, allogeneic transplantation

## Abstract

**Background:**

Therapy-related myeloid neoplasms (t-MN) are aggressive hematologic malignancies with poor prognosis and high-risk clinical features. Recent advances have highlighted the role of molecular data in refining prognostic models. This study aims to analyze a monocentric cohort of t-MN patients, focusing on the clinical and prognostic impact of prior malignancies and their associated molecular landscape.

**Methods:**

A retrospective analysis was conducted on 61 patients diagnosed with t-MN from an Oncology Hospital and referred to a hematology Unit. Diagnoses were based on established criteria for therapy-related myelodysplastic syndrome (t-MDS) and therapy-related acute myeloid leukemia (t-AML), with a history of prior exposure to cytotoxic therapy. Cytogenetic and molecular analyses supported the diagnoses. Risk stratification was performed using the revised International Prognostic Scoring System (IPSS-R) and molecular IPSS (IPSS-M) for t-MDS and the 2022 European LeukemiaNet (ELN) classification for t-AML.

**Results:**

Overall, 61 patients with t-MN were diagnosed: 38 (62.3%) with t-MDS, and 23 (37.7%) with t-AML. The median latency from primary cancer to t-MN diagnosis was 5.8 years (IQR: 2.6–12.5). Risk stratification identified 63.2% of t-MDS cases as IPSS-R very-low to intermediate risk, while 57.9% were reclassified as IPSS-M moderate-high to very high risk. Patients with prior hematologic cancer showed a greater tendency toward higher IPSS-R (*p*=0.021) and IPSS-M (*p*=0.015) risk compared to solid cancer. The IPSS-M, more accurately than R-IPSS, demonstrated predictive value for survival in both univariate and multivariate analyses and effectively predicted leukemic progression in t-MDS. *TP53*-mutated cases were more prevalent in patients with prior hematologic cancer (*p*=0.043) and associated with longer latency (8.2 years) compared to *TP53* wild type (6.1 years, *p*=0.044). Allogeneic transplantation proved beneficial, significantly improving survival outcomes in eligible t-MDS and t-AML patients.

**Conclusions:**

t-MN exhibits distinct clinical and molecular profiles according to prior malignancy type. Intriguingly, our analysis reveals a distinct latency pattern in *TP53*-mutated cases, suggesting unique leukemogenic dynamics. Moreover, IPSS-M proved highly accurate in predicting t-MDS survival. Integrating molecular data into prognostic models enhances risk stratification and informs therapeutic strategies, potentially improving outcomes for t-MN patients. Further studies are needed to validate these findings and refine tailored treatment approaches.

## 1 Introduction

Therapy-related myeloid neoplasms (t-MN), comprised of therapy-related myelodysplastic syndromes (t-MDS), acute myeloid leukemia (t-AML), and myelodysplastic/myeloproliferative neoplasms (t-MDS/MPN), represent a rare but severe complication of cytotoxic treatments used for both malignant and non-malignant diseases (1, 2). While these treatments have substantially improved survival rates in cancer patients, they are also associated with an increased risk of secondary hematologic malignancies (3).

Cytogenetic and molecular profiling have revealed significant differences between t-MN and *de novo* MN. Specifically, therapy-related cases are characterized by a higher prevalence of high-risk cytogenetic abnormalities, including complex karyotypes (4, 5). In addition, next-generation sequencing (NGS) has revealed a mutational profile that, while largely shared with *de novo* cases, demonstrates a higher prevalence of mutations in adverse prognostic genes such as *TP53*, *SETBP1*, and *SRSF2*, and a lower incidence of mutations in genes like *NPM1*, *FLT3*, and *IDH1*/*2* (6).

Recent advancements have integrated molecular data into classification and prognostic models for both t-MDS and t-AML. In t-MDS, a major achievement has been the development of the molecular International Prognostic Scoring System (IPSS-M), which incorporates molecular information and the revised IPSS (IPSS-R). The original study included 8% of t-MDS cases, which were more frequently categorized as IPSS-M high/very high risk but exhibited comparable outcomes to *de novo* cases within each risk category (7). Similarly, t-AML has not consistently emerged as an independent risk factor for survival in some studies. In contrast, others report worse outcomes in intermediate-high risk categories, with no difference in favorable risk compared to *de novo* AML (5, 8).

Historically, the prognosis of t-MN has been poor, with a 5-year survival rate of approximately 10% (9). This outcome is primarily influenced by factors such as advanced age, a higher burden of comorbidities, and the cumulative toxicities of prior cytotoxic therapies (10). To date, molecular analysis has been evaluated, providing prognostic insights and highlighting significant differences based on the latency from the primary malignancy (4). However, limited data are available regarding the molecular profile in relation to the primary cancer.

This study investigates the clinical and molecular characteristics of a monocentric cohort of t-MN patients from an Oncology Hospital referred to the Hematology Unit, focusing on the prognostic implications of prior malignancy types and their associated molecular profiles. These findings may enhance current risk assessment models and guide the development of personalized therapeutic strategies, ultimately improving patient outcomes.

## 2 Materials and methods

### 2.1 Study population

We performed a retrospective analysis of 61 patients diagnosed with t-MN and treated at our institution between January 2009 and November 2024. Clinical data were retrieved from medical records. The diagnosis of t-MN was established based on the recognized criteria for MDS or AML, combined with a documented history of exposure to cytotoxic therapy for unrelated malignancies (2). Patients with MN and a history of prior malignancy were excluded if they had not received chemotherapy or radiotherapy, immunotherapy, hormone therapy, target therapy, or if they had undergone surgery alone. Bone marrow evaluations were conducted using cytology, histology, and flow cytometry. The general clinical condition of patients was assessed using the Eastern Cooperative Oncology Group (ECOG) performance status (PS) (11). For t-MDS, risk classification was based on both the IPSS-R (12) and IPSS-M (7). For t-AML, risk stratification followed the 2022 ELN guidelines (13). The SIE/SIES/GITMO criteria were retrospectively applied to categorize patients as fit or unfit for intensive chemotherapy (14).

### 2.2 Cytogenetic and genetic analysis

Chromosomal banding was performed using standard techniques, with karyotypes described according to the International System for Human Cytogenetic Nomenclature (ISCN). Mutational analysis of *FLT3-ITD*, *RUNX1::RUNX1T1*, *CBF::MYH11*, *PML::RARA*, *WT1*, *NPM1*, and *BCR::ABL1* was conducted using real-time PCR (RT-PCR).

Targeted NGS was performed to analyze 40 genes frequently mutated in myeloid neoplasms. Genomic DNA extracted from peripheral blood or bone marrow was processed using the Oncomine Myeloid Research panel (Thermo Fisher Scientific) and sequenced on the Ion Torrent GeneStudio™ S5 system with a targeted coverage depth exceeding 300x. Data analysis was conducted using Torrent Suite and Ion Reporter™ Software, with reads aligned to the hg19 reference genome. Variants were reported if the variant allele frequency (VAF) was ≥5% with ≥300x coverage or ≥2% for hotspot mutations with ≥100x coverage. Only pathogenic, likely pathogenic, or variants of uncertain significance were reported, adhering to HGVS nomenclature guidelines (15). Polymorphisms and benign variants were excluded from the report.

### 2.3 Statistical analysis

Continuous variables were reported as medians with interquartile ranges (IQR), and categorical variables as frequencies and percentages. Comparisons between groups were conducted using Chi-squared tests for nominal variables, Fisher’s exact test, and the Wilcoxon-Mann-Whitney test for non-parametric data. Logistic regression was employed to calculate odds ratios (ORs) with 95% confidence intervals (95% CI). Overall survival (OS) was defined as the time from t-MN diagnosis to death or last follow-up, and progression-free survival (PFS) was defined as the time from t-MDS diagnosis to diagnosis of leukemic progression. Kaplan-Meier curves were used for survival analysis, with differences assessed via the Log-Rank test. Hazard ratios (HR) with 95% CI for survival-associated factors were calculated using univariate and multivariate Cox proportional hazards regression. A *p*-value < 0.05 was considered statistically significant. Statistical analyses were conducted using R software (R Core Team, 2021), version 4.1.2 (R Foundation for Statistical Computing, Vienna, Austria).

## 3 Results

### 3.1 Demographics and biological features of t-MN cohort

Patient clinical and biological characteristics are detailed in **Table 1**. Overall, the analysis included 61 patients with t-MN, comprising 38 patients (62.3%) diagnosed with t-MDS and 23 (37.7%) with t-AML. The median age at t-MN diagnosis was 69.9 years (IQR: 59.0–74.6). Notably, patients with t-MDS were significantly older than t-AML patients at t-MN diagnosis (*p*<0.001). The majority of patients exhibited PS ECOG of 0–1 (86.9%), with no significant difference between t-MDS and t-AML subgroups (*p*=0.784). At the time of t-MN diagnosis, 38 patients (48.2%) presented with at least one comorbidity, most frequently cardiovascular disease (46.4%). Comorbidity burden and blood count data were comparable between diagnostic subgroups, except for a significantly lower median platelet count observed in t-AML (*p*=0.033).

**Table 1:** Clinical features at diagnosis of t-MN, including differences between t-MDS and t-AML.

Clinical features	Total cohort n=61	t-MDS n=38	t-AML n=23	*p*†
**Male sex, n (%)**	28 (45.9)	19 (50.0)	10 (33.5)	0.975
**Age, median years (IQR)**	69.9 (59.0-74.6)	72.1 (67.5-79.5)	62.5 (49.4-68.8)	**<0.001**
**PS ECOG**				0.784
0-1	53 (86.9)	33 (82.3)	20 (81.8)	
2-3	8 (12.7)	4 (17.7)	4 (18.1)	
**Comorbidities at diagnosis, n (%)**	41 (67.2)	27 (71.1)	14 (60.9)	0.589
CV history, n (%)	26 (46.4)	18 (52.9)	8 (36.3)	0.346
Type 2 Diabetes, n (%)	8 (14.2)	6 (17.6)	2 (9.0)	0.615
Chronic kidney disease, n (%)	5 (8.9)	4 (11.7)	1 (4.5)	0.655
**Cancer history, n (%)**				
Solid tumor	38 (60.6)	23 (64.7)	15 (68.2)	0.983
Hematologic malignancy	23 (36.1)	15 (34.2)	8 (34.8)	0.915
**Hemoglobin, g/dL, median (IQR)**	8.9 (8.0.4)	9.0 (8.0-9.7)	8.4 (7.7-9.1)	0.082
**Leukocyte count, x10^9^/L, median (IQR)**	2.7 (2.0-5.1)	3.0 (2.3-4.3)	2.3 (1.8-8.3)	0.774
ANC, x10^9^/L, median (IQR)	1.3 (0.6-2.3)	1.4 (0.9-2.0)	0.8 (0.2-3.2)	0.341
**Platelet count, x10^9^/L, median (IQR)**	74 (37.8-161.5)	92 (44.8-274.0)	55 (30.8-116.5)	**0.033**
**Bone marrow blast, %, median (IQR)**	10 (2.5-28)	3 (2-8)	45 (20-70)	
**Median time from primary cancer diagnosis, years (IQR)**	5.8 (2.6-12.5)	7.6 (2.7-11.6)	4.1 (2.1-13.5)	0.413
Solid tumor	5.0 (2.5-12.4)	4.5 (2.3-10.9)	5.7 (3.0-14.6)	0.586
Hematologic malignancy	6.5 (4.1-15.6)	5.4 (2.7-11.3)	2.9 (1.8-5.6)	0.441

ANC, absolute neutrophil count; CV, cardiovascular; IQR, interquartile range; PS ECOG, performance status according to Eastern Cooperative Oncology Group; t-AML, therapy-related acute myeloid leukemia; t-MDS, therapy-related myelodysplastic syndrome; t-MN, therapy-related myeloid neoplasia.

†*p* values indicate differences between t-MDS and t-AML.Bold values reported in the "p" column indicate statistical significance.

**Figure 1:**
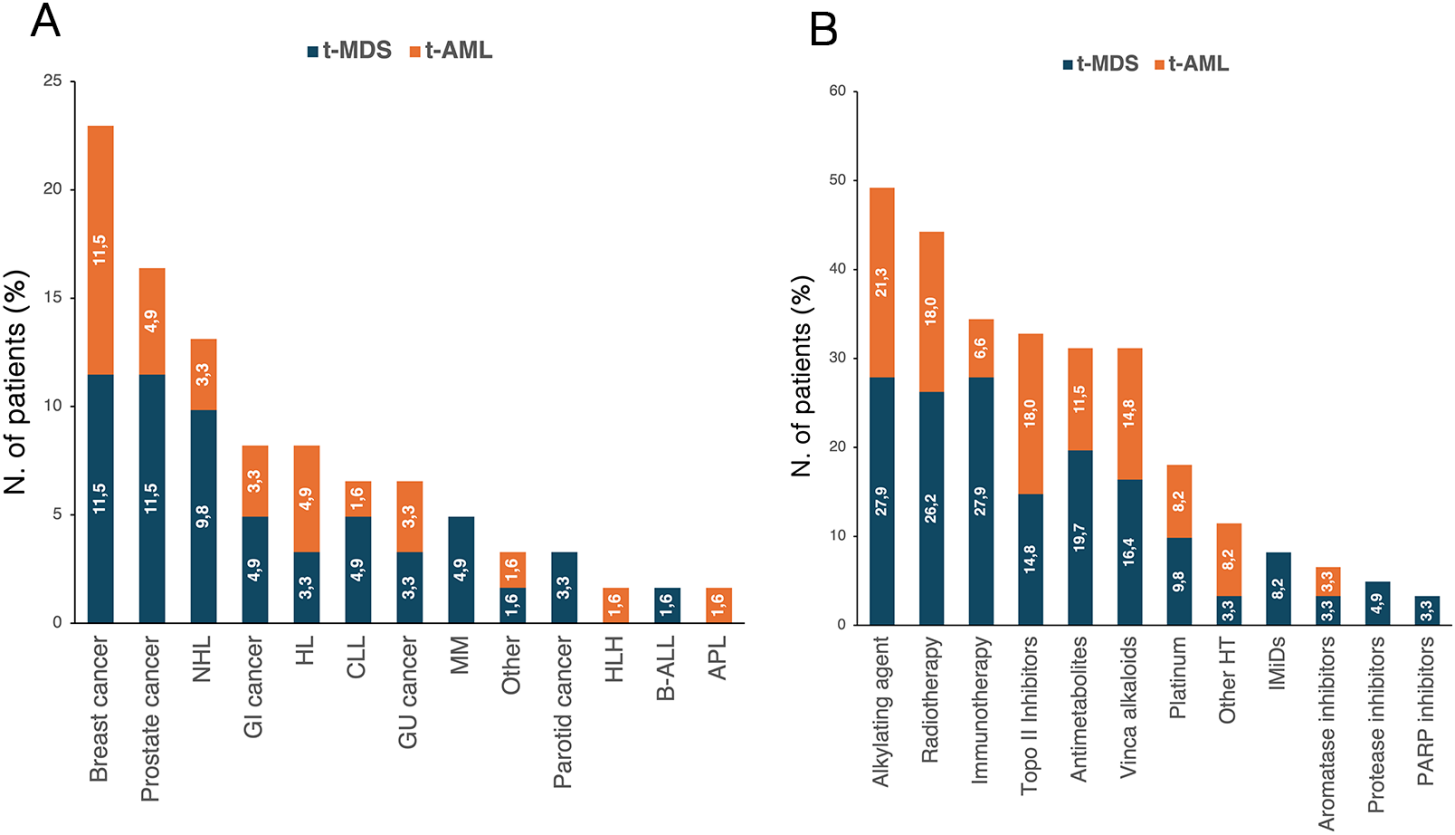
Distribution of primary cancer diagnoses **(A)** and prior cytotoxic therapies **(B)** according to diagnosis of t-MDS and t-AML. The percentages reported within the bars were calculated based on the total number of patients with t-MN (n=61). APL, acute promyelocytic leukemia; B-ALL, Acute lymphoblastic leukemia B; CLL, chronic lymphocytic leukemia; GI, gastrointestinal; GU, genitourinary; HL, Hodgkin lymphoma; HLH, hemophagocytic lymphohystiocytosis; HT, hormone therapy; IMiDs, immunomodulatory drugs; MM, multiple myeloma; NHL, non-Hodgkin lymphoma; t-AML, therapy-related acute myeloid leukemia; t-MDS, therapy-related myelodysplastic syndrome; t-MN, therapy-related myeloid neoplasms.

A prior history of solid and hematologic cancer was documented in 38 (62.3%) and in 23 (37.7%) patients, respectively. The spectrum of primary malignancies and previous cytotoxic therapies is detailed in **Figure 1**. Cytogenetic analysis was conducted in all enrolled patients (**Supplementary Figure 1**), revealing abnormalities in 37 cases (60.7%), including a complex karyotype in 11 (18%). Notably, complex karyotypes were more frequent in patients with prior hematologic malignancies than in those with solid tumors (*p*=0.049). No other significant cytogenetic differences were observed based on cancer history.

Overall, 31 (50.8%) also underwent NGS analysis (**Supplementary Figure 1**). Among the 23 patients with NGS-detected mutations, 12 (52.2%) harbored a single isolated variant, 7 (30.4%) exhibited two co-occurring mutations, and 4 (17.4%) carried three or more concurrent mutations. Subgroup analysis by primary cancer type showed no significant difference in the frequency of isolated versus co-occurring mutations (*p*=0.931). However, *TP53* mutations were more prevalent in patients with hematologic malignancies (n=6) compared to those with solid tumors (n=1) (*p*=0.043). Notably, none of the *TP53*-mutated cases harbored *DNMT3A*, *TET2*, or *ASXL1* (DTA) mutations. In contrast, DTA co-mutations with non-DTA genes were observed in 7 cases (30.4%), more commonly in patients with prior solid tumors than in those with hematologic malignancies, though this difference did not reach statistical significance (*p*=0.619).

**Figure 2:**
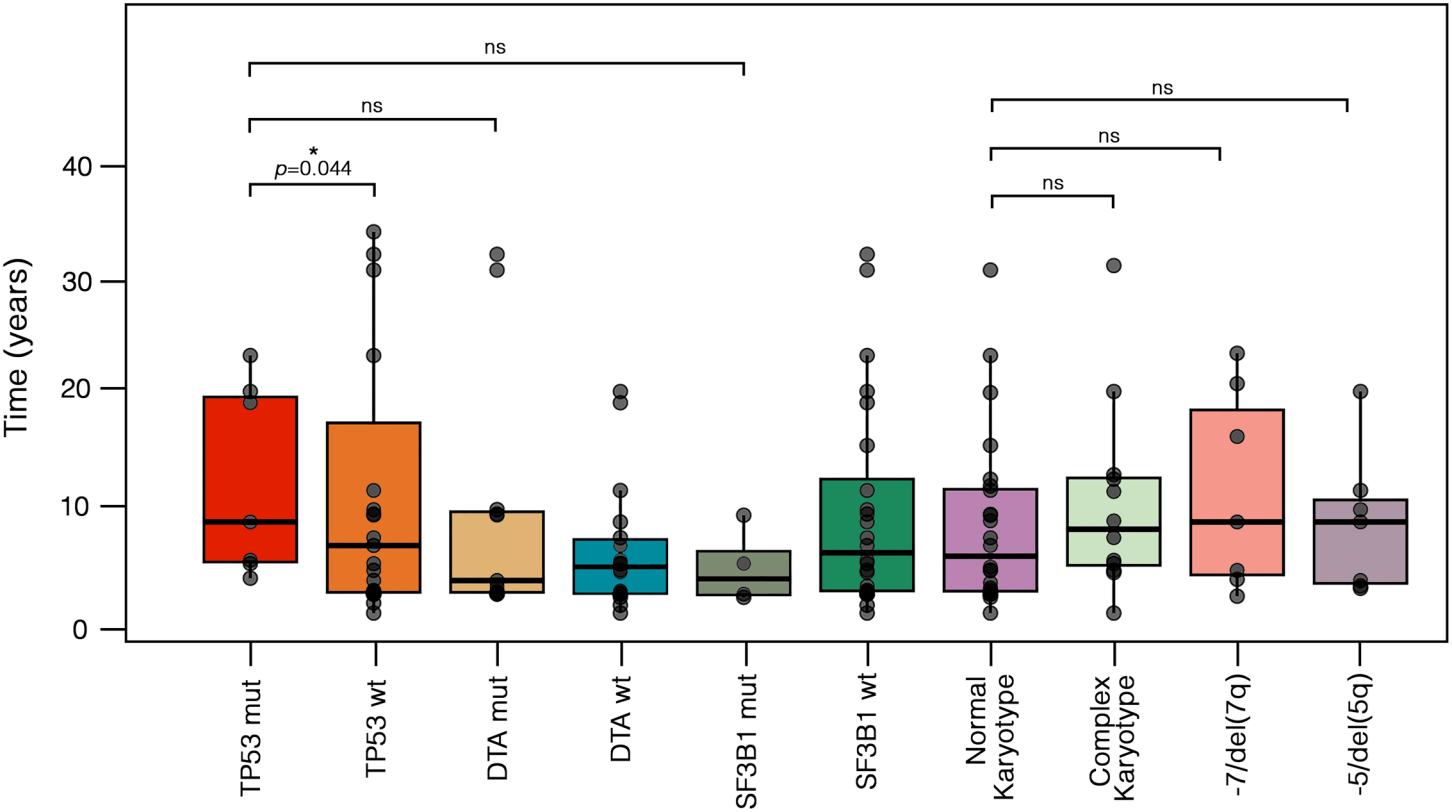
Different latencies from primary cancer to t-MN according to cytogenetic and molecular abnormalities. Boxplots display the distribution of latency times, with individual values, median, and outliers shown for each molecular and cytogenetic subgroup. Statistical differences were assessed using the Kruskal-Wallis test. *TP53*-mut cases exhibited a longer median latency compared to *TP53*-wt [8.2 years (IQR: 4.6–19.3) vs. 6.1 years (IQR: 1.9–17.0), *p* = 0.044]. In contrast, no significant differences were observed when comparing *TP53*-mut cases to DTA-mut [3.0 years (IQR: 1.9–9.1), *p* = 0.23] or *SF3B1*-mut cases [3.1 years (IQR: 1.7–5.6), *p* = 0.450]. Similarly, no significant differences were found between cases with a normal karyotype [4.2 years (IQR: 1.9–11.0)] and those with a complex karyotype [7.5 years (IQR: 4.3–12.1), *p* = 0.466], -7/del(7q) [8.2 years (IQR: 3.5–18.2), *p* = 0.429], or -5/del(5q) [8.2 years (IQR: 2.8–10.2), *p* = 0.315]. DTA, *DNMT3A*, *ASXL1* and *TET2*; IQR, interquartile range; mut, mutated; ns, not significative; t-MN, therapy-related myeloid neoplasms; wt, wild-type.

The median latency from primary malignancy to t-MN was 5.8 years (IQR: 2.6–12.5), with no significant differences between t-MDS and t-AML subgroups (*p*=0.413). Similarly, latency did not differ significantly between patients with a prior history of solid tumors and those with hematologic malignancies (*p*=0.536). To further characterize latency patterns, we stratified patients into three groups based on previously published data (16): short latency (<1 year), intermediate latency (1–10 years), and long latency (>10 years) (**Supplementary Figure 1**). The majority of cases (n=38, 62.3%) exhibited intermediate latency, while 18 (29.5%) had long latency and 5 (8.2%) showed short latency. Patients with a prior hematologic malignancy were more likely to experience short latency compared to those with solid tumors (17.4% vs. 2.6%, *p*=0.0412). No significant differences emerged for intermediate (56.5% vs. 65.8%, *p*=0.556) or long latency (26.1% vs 31.6%, *p*=0.868). Notably, *TP53* mutations, classified as either *single-hit* (n=3) or *multi-hit* (n=4) according to ICC criteria (1), have shown a longer median latency from primary disease compared to *TP53* wild-type [8.2 years (IQR: 4.6–19.3) vs. 6.1 years (IQR: 1.9–17.0), *p*=0.044] (**Figure 2**). Conversely, no significant differences were observed between DTA-mutated cases compared to *TP53* or *SF3B1* mutated. No differences were instead recorded between normal and complex karyotype, -7/del(7q) and -5/del(5q) cohort (**Figure 2**).

### 3.2 Clinical features and risk stratification of t-MDS patients

**Figure 3:**
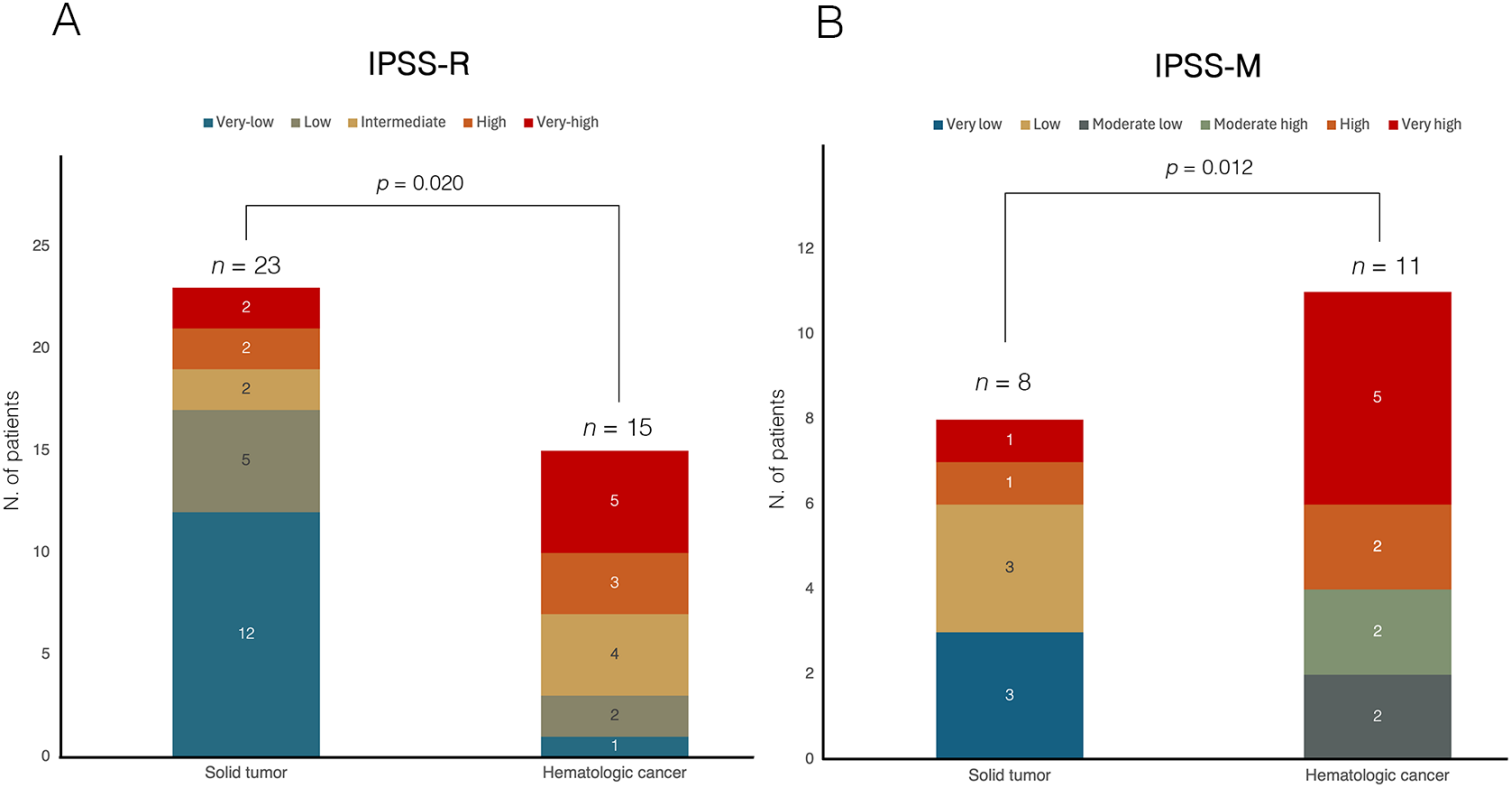
Comparison of risk stratification according to **(A)** IPSS-R and **(B)** IPSS-M in t-MDS cohort, categorized by prior history of solid or hematologic malignancy. In both panels, bars represent the two groups (solid tumor vs hematologic cancer), with internal segments corresponding to individual risk categories. The numbers within each segment indicate the absolute number of patients per risk category. In panel **(A)**, based on the IPSS-R classification, among patients with a history of solid tumors (n=23), 12 patients (52.2%) were classified as very low risk, 5 (21.7%) as low risk, 2 (8.7%) as intermediate risk, and 4 (17.4%) as high or very high risk. In contrast, among patients with prior hematologic malignancies (n=15), 1 patient (6.7%) were categorized as very low risk, 2 (13.3%) as low risk, 4 (26.7%) as intermediate risk, and 8 patients (53.3%) as high or very high risk, indicating a significantly higher proportion of patients in the higher-risk categories in this group (*Chi*-square test, *p* = 0.020). In panel **(B)**, among patients evaluable for IPSS-M (n=19), those with a history of solid tumors (n=8) were stratified as very low in 3 patients (37.5%), low in 3 patients (37.5%), and high or very high in 2 patients (25.0%). Conversely, patients with prior hematologic malignancies (n=11) were classified as moderate-low in 2 patients (18.2%), moderate-high in 2 patients (18.2%), and high or very high in 7 patients (63.6%), demonstrating a significantly greater representation in the higher-risk categories (*Chi*-square test, *p* = 0.012). IPSS-M, molecular international prognostic scoring system; IPSS-R, revised IPSS; t-MDS, therapy-related myelodysplastic syndrome.

According to the 2022 WHO classification, 12 patients (31.6%) were defined as MDS with low blasts (MDS-LB), 7 patients (18.4%) as MDS with increased blasts-1 (MDS-IB1), 6 patients (15.8%) as MDS with increased blasts-2 (MDS-IB2), 5 patients (13.2%) as MDS with *TP53* mutation (*TP53*-MDS), 3 patients (7.9%) as MDS with del(5q) (MDS-5q), 3 patients (7.9%) as MDS with *SF3B1* mutation, and one patient (2.6%) as MDS with fibrosis. According to the IPSS-R score, most patients were categorized as very-low, low, and intermediate risk (63.2%) (**Figure 3**). Nineteen patients (50.0%) were evaluable for the IPSS-M score; of these, 57.9% had an IPSS-M risk ranging from moderate-high to very high. Regarding the primary cancer diagnosis, 23 patients (60.5%) had a history of solid tumors, and 15 (39.5%) had a previous hematologic malignancy. The distribution of primary tumor types among t-MDS patients is shown in **Figure 1**. Notably, patients with a history of hematologic cancer were younger (*p*=0.003), had lower platelet counts (*p*=0.002), and had a higher blast percentage at diagnosis (*p*=0.021) compared to patients with a history of solid tumors (**Table 2**). Consequently, a greater tendency toward higher-risk IPSS-R (*p*=0.020) and IPSS-M (*p*=0.012) categories was observed in patients with a history of hematologic malignancy. However, the comparison of latency times was not significant (*p*=0.347).

**Table 2:** Key features of t-MDS and comparison between patients with a history of solid tumors and hematologic malignancies. .

Clinical features	Total cohort n= 38	Solid tumor n= 23	Hematologic cancer n= 15	*p*†
**Male sex, n (%)**	19 (50.0)	10 (43.5)	9 (60.0)	0.503
**Age, median years (IQR)**	72.1 (67.5-79.5)	77.2 (72.9-80.1)	67.9 (55.1-71.2)	**0.003**
**Hemoglobin, g/dL, median (IQR)**	9.0 (8.0-9.7)	9.1 (8.0-9.7)	8.5 (7.9-9.4)	0.319
**Leukocyte count, x10^9^/L, median (IQR)**	3.0 (2.3-4.3)	3.3 (2.3-4.5)	2.6 (2.0-4.1)	0.580
ANC, x10^9^/L, median (IQR)	1.4 (0.9-2.0)	1.8 (1.0-2.1)	1.3 (0.7-1.5)	0.157
**Platelet count, x10^9^/L, median (IQR)**	92 (44.8-274.0)	232 (89-294)	50 (34-66)	**0.002**
**Bone marrow blast, %, median (IQR)**	3 (2-8)	2 (1.0-7.0)	8 (3.0-15.0)	**0.021**
**Median latency to t-MDS, years (IQR)**	7.6 (2.7-11.6)	4.5 (2.3-10.9)	5.4 (2.7-11.3)	0.347
**N. of patients with karyotype abnormalities, n (%)**	20 (55.3)	8 (36.4)	12 (75.0)	**0.042**
**Progression to AML, n (%)**	9 (23.7)	3 (14.3)	5 (33.3)	0.114
**Median time to progression to AML, months (IQR)**	4.6 (2.3-9.6)	30.3 (27.6-83.3)	4.6 (3.6-7.5)	0.142

AML, acute myeloid leukemia; ANC, absolute neutrophil count; IQR, interquartile range; t-MDS; therapy-related myelodysplastic syndrome.

†*p* values indicate differences between t-MDS and t-AML.Bold values reported in the "p" column indicate statistical significance.

### 3.3 Cytogenetic and molecular profile of the t-MDS cohort

**Figure 4:**
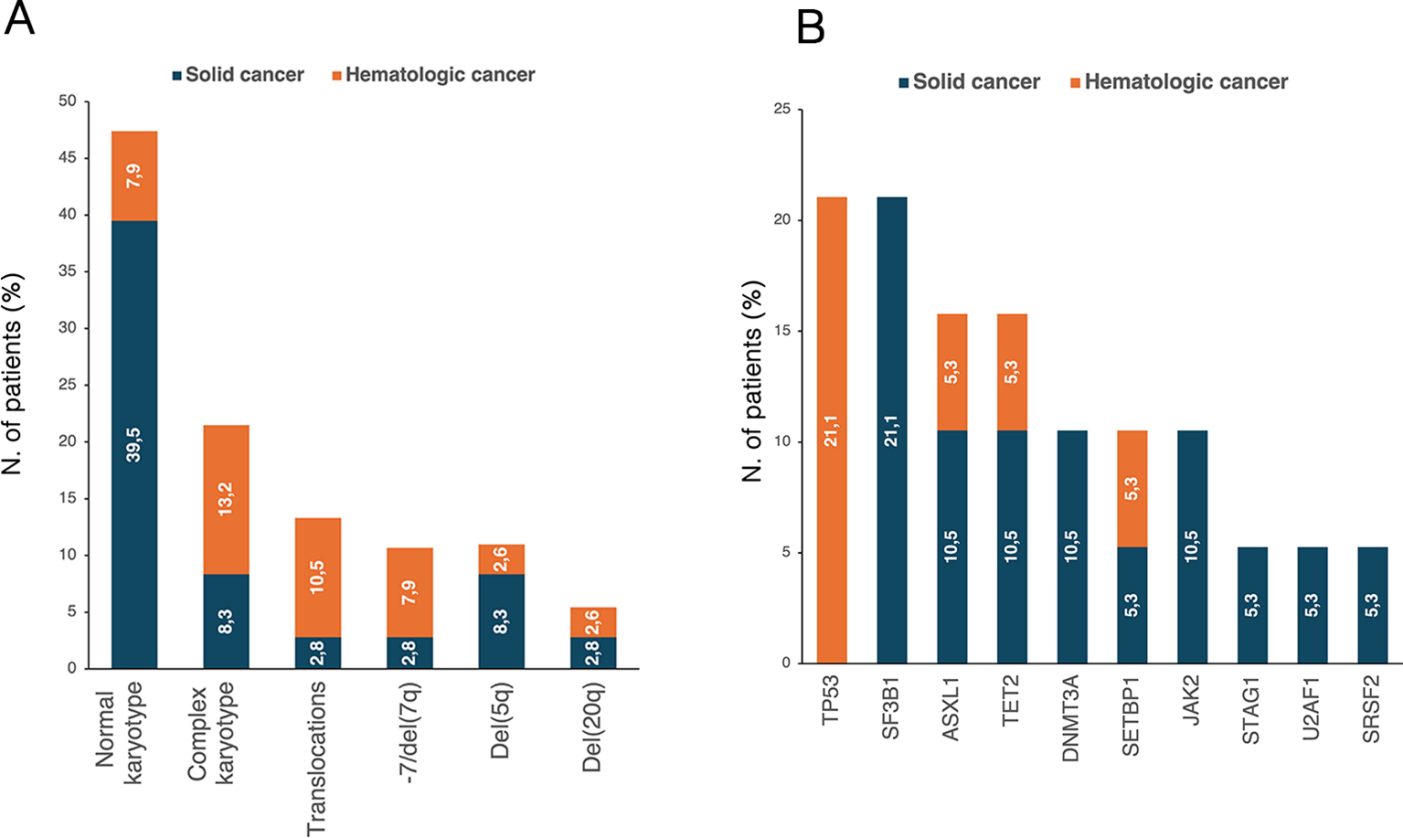
Distribution of **(A)** karyotype abnormalities in t-MDS patients with available cytogenetic data (n=36) and **(B)** NGS-detected mutations in patients who underwent NGS analysis (n=19). The percentages reported within the bars were calculated based on the total number of patients in each respective group. NGS, next-generation sequencing; t-MDS, therapy-related myelodysplastic syndrome.

Cytogenetic abnormalities (**Figure 4A**) were reported in 20 patients (52.6%). Of these, 11 patients (32.4%) had two concomitant abnormalities, and 8 patients (23.5%) had ≥3 abnormalities. According to cancer history, patients with prior hematologic neoplasm had a higher frequency of detectable cytogenetic abnormalities than those with a history of solid cancer (*p*=0.042). Nineteen patients (50%) underwent NGS analysis, and mutations were detected in 15 patients (78.9%). Among them, 10 patients (66.7%) had a normal karyotype. Overall, *TP53*, *SF3B1, ASXL1* and *TET2* were the most frequently identified abnormalities (**Figure 4B**). DTA mutations were found in 42.1% of patients, more frequently in the solid cancer group than in the hematologic cancer group, though not significantly (50.0% vs. 25.0%, *p*=0.349). In our cohort, DTA mutations did not significantly correlate with age (*p*=0.861). All four patients with *TP53*-MDS had a history of hematologic malignancy, and 75% had a complex karyotype. Additionally, younger patients were at higher risk of *TP53* alterations [OR 0.87 (95% CI, 0.72-0.99), *p*=0.040].

### 3.4 Treatment and survival analysis in t-MDS patients

Overall, 26 patients (68.4%) received treatment for t-MDS, including recombinant erythropoietin in 14 patients (36.8%), hypomethylating agents (HMA) in 8 patients (21.1%), eltrombopag in two patients (5.3%), luspatercept and lenalidomide in one patient each. Among patients treated with HMA, a median of 2 cycles was administered, with more than 50% of patients receiving ≤3 treatment cycles. Three patients who received HMA were referred for HSCT after achieving complete response (CR), with a 4-year OS of 100% compared to 25% (95% CI, 0.1-0.6) for non-transplanted patients. Of the remaining patients, two died of leukemic progression, and two of infectious complications.

**Figure 5:**
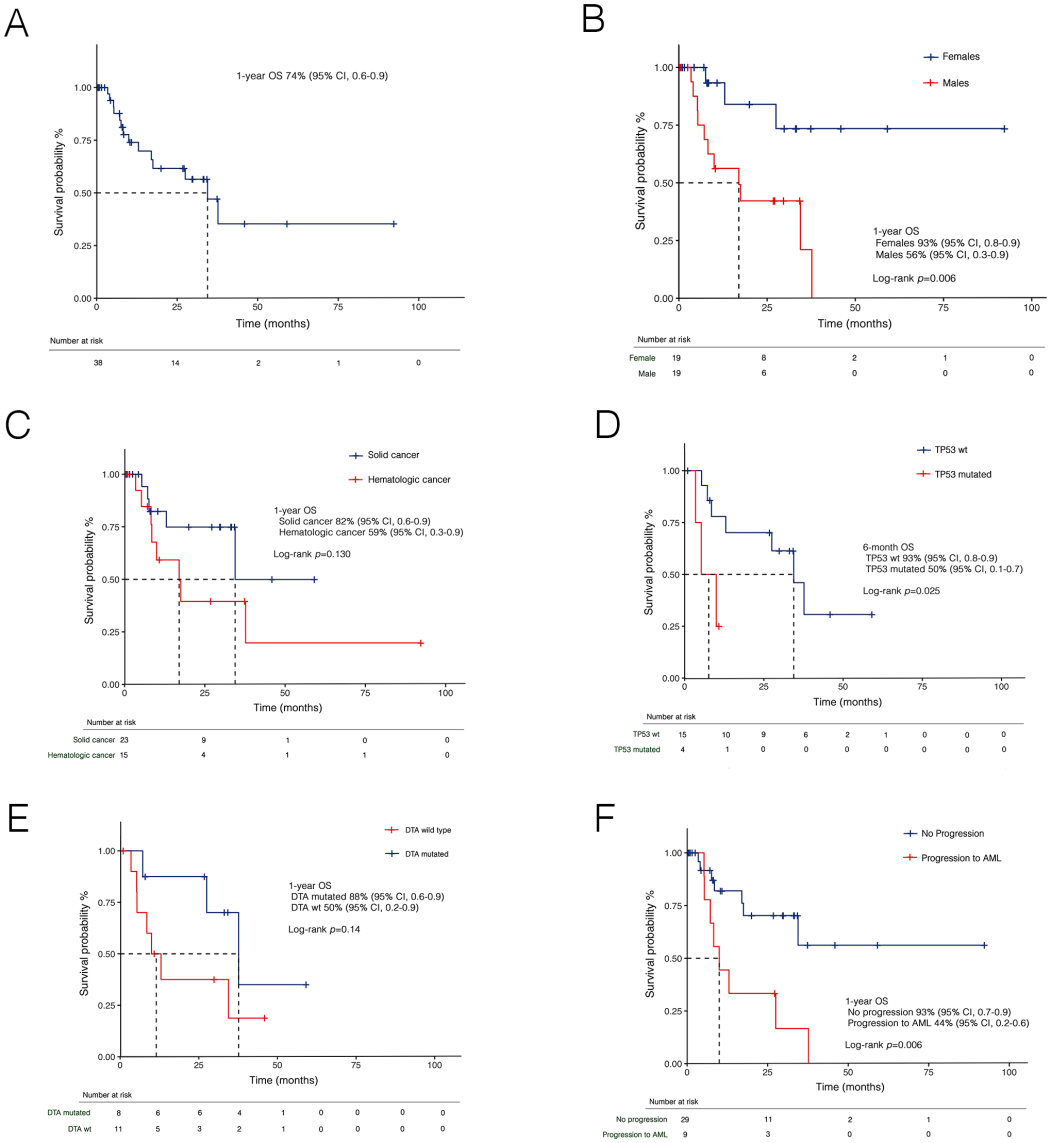
Kaplan-Meier curves for overall survival (OS) in t-MDS in the whole cohort **(A)**, and stratified by sex **(B)**, prior malignancies **(C)**, *TP53* **(D)**, DTA mutational status **(E)**, and disease progression **(F)**. AML, acute myeloid leukemia; DTA, *DNMT3A, TET2* and *ASXL1*; t-MDS, therapy-related myelodysplastic syndrome; wt, wild-type.

With a median follow-up of 10.6 months (IQR: 5.7–29.8), the 1-year OS was 74% (95% CI, 0.6–0.9) (**Figure 5A**). Stratified analysis by sex revealed a significantly lower survival in male patients (**Figure 5B**), with a 1-year OS of 56% (95% CI, 0.3-0.8) compared to 93% (95% CI, 0.8-0.9) in females (*p*=0.006). Patients with a history of hematologic cancer had a 1-year OS of 59% (95% CI, 0.3–0.9) compared to 82% (95% CI, 0.6–0.9) in those with prior solid tumors, though without statistical significance (*p*=0.130) (**Figure 5C**). Notably, a significantly lower 6-month OS was found also for *TP53*-MDS compared to *TP53* wild-type MDS (*p*=0.025) (**Figure 5D**). Conversely, no significant differences were observed based on the DTA mutation status (**Figure 5E**).

### 3.5 Progression rates to t-AML and prognostic performance of risk score

**Figure 6:**
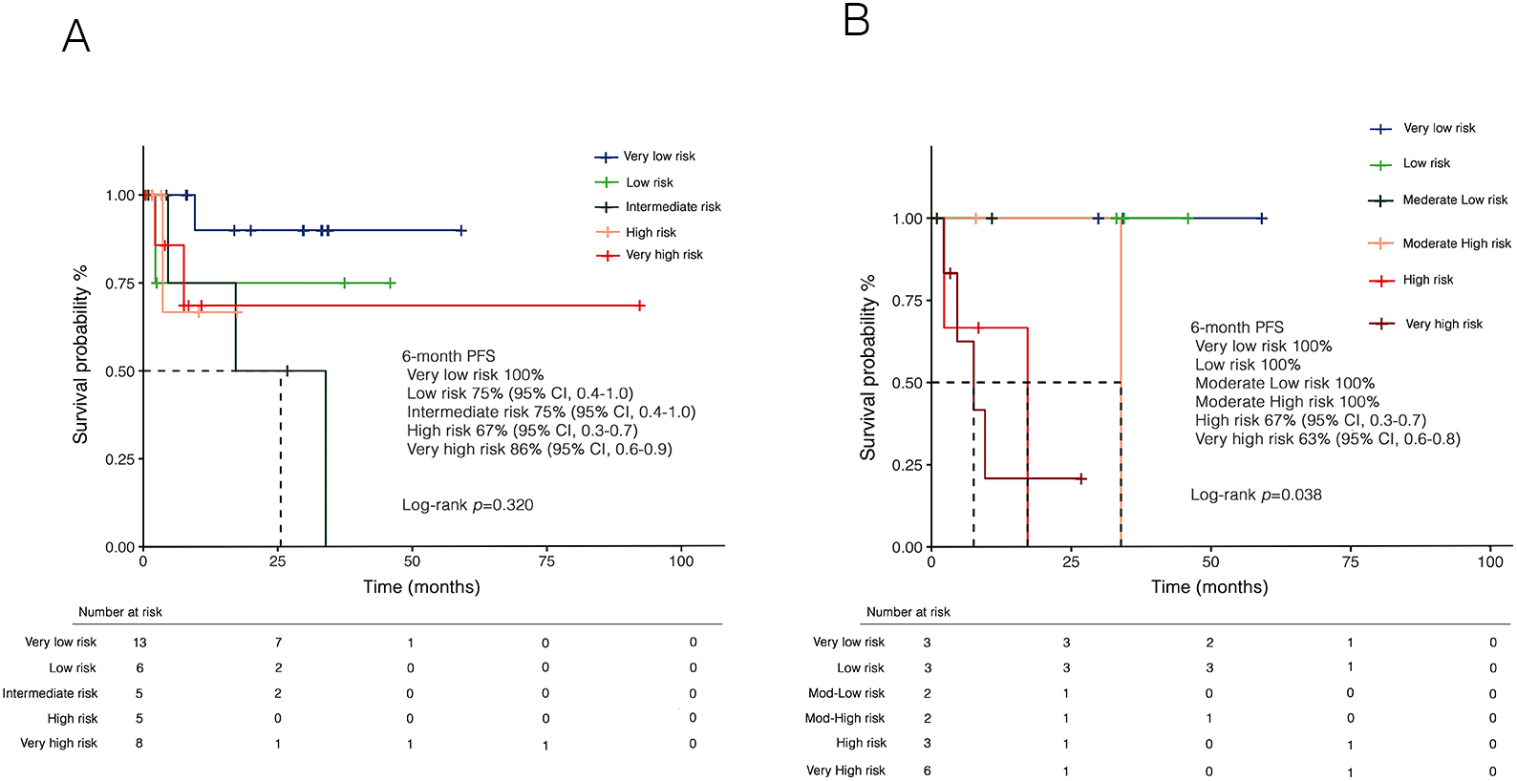
Kaplan-Meier curves for progression-free survival (PFS) according to **(A)** IPSS-R and **(B)** IPSS-M. Panel **(A)** shows PFS stratified by IPSS-R risk categories, with no significant differences observed between cohorts in 6-month PFS (*p*=0.320). Panel **(B)** presents PFS stratified by IPSS-M, demonstrating a statistically significant difference between risk categories in 6-month PFS (*p*=0.038). IPSS-M, molecular international prognostic scoring system; IPSS-R, revised IPSS.

Overall, 9 patients (23.7%) experienced progression to AML. At a median follow-up of 9.0 months (IQR: 3,5-29.8), the 1-year PFS was 79% (95% CI, 0.65-0.96). PFS was lower in patients with prior hematologic cancer compared to solid tumors [64% (95% CI, 0.4-0.8) vs 87% (0.7-0.9)], with a trend towards statistical significance (*p*=0.071). Expectedly, disease progression was associated with a dismal prognosis (**Figure 5F**). In univariate analysis (**Table 3**), female sex was associated with better survival, while disease progression was associated with worse outcomes. Additionally, both the IPSS-R (*p*=0.020) and IPSS-M (*p*=0.012) scores were predictive of survival. However, in multivariate analysis, only IPSS-M remained a predictive factor for survival (HR 2.9, 95% CI 1.06-8.4; *p*=0.038). Notably, no differences were found in terms of 6-month PFS between risk cohorts according to IPSS-R (*p*=0.320) (**Figure 6A**). At the same time, IPSS-M effectively predicted progression to AML among different risk classes (*p*=0.038) (**Figure 6B**).

**Table 3:** Predictive factors for survival in t-MDS in univariate and multivariate analysis.

Variables	Univariate analysis			Multivariate analysis		
	HR	95% CI	*p*	HR	95% CI	*p*
**Female sex**	0.2	0.05-0.7	**0.013**	0.19	0.01-2.6	0.218
**IPSS-R**	1.5	1.1-2.1	**0.021**	1.1	0.8-1.6	0.507
**IPSS-M**	2.7	1.3-6.8	**0.013**	2.9	1.06-8.4	**0.038**
**Hematologic cancer history**	2.8	0.93-6.9	0.066	0.52	0.08-3.1	0.481
**Progression to AML**	4.4	1.5-5.7	**<0.001**	1.6	0.2-6.7	0.604

AML, acute myeloid leukemia; CI, confidence intervals; HR, hazard ratio; IPSS-M, molecular international prognostic scoring system; IPSS-R, revised IPSS.Bold values reported in the "p" column indicate statistical significance.

### 3.6 Clinical and genetic features of t-AML

According to the ELN 2022 classification, three patients (13.0%) were considered at favorable risk, 6 patients (26.1%) at intermediate risk, and 11 patients (47.8%) at adverse risk. Three patients (13.0%) had a diagnosis of APL and were all considered at low risk according to Sanz’s risk score. A history of solid and hematologic cancer was reported in 15 (65.2%) and 8 patients (34.8%), respectively. No differences were observed between the two groups regarding sex, age, and fitness (**Table 4**). Additionally, median latency from primary cancer was shorter for patients with a history of hematologic tumors, although not statistically significant (*p*=0.227) (**Table 4**). The distribution of primary diagnoses and treatments received for the primary tumor are shown in **Figure 1**.

**Table 4:** Key features of t-AML and comparison between patients with a history of solid tumors and hematologic malignancies.

Clinical features	Total cohort n= 23	Solid tumor n= 15	Hematologic cancer n= 8	*p*†
**Male sex, n (%)**	9 (39.1)	4 (26.7)	5 (62.5)	0.219
**Age, median years (IQR)**	60.6 (48.9-68.2)	64.5 (53.6-69.1)	57.2 (44.2-67.3)	0.282
**Unfit patient, n (%)**	6 (26.1)	4 (26.7)	2 (25.0)	0.680
**Hemoglobin, g/dL, median (IQR)**	8.4 (7.7-9.1)	8.3 (7.3-9.0)	9.1 (8.4-10.6)	0.208
**Leukocyte count, x10^9^/L, median (IQR)**	2.3 (1.8-8.3)	3.6 (1.8-13.9)	2.0 (1.6-2.8)	0.518
ANC, x10^9^/L, median (IQR)	0.8 (0.2-3.2)	0.8 (0.4-4.1)	0.6 (0.1-1.7)	0.986
**Platelet count, x10^9^/L, median (IQR)**	55 (30.8-116.5)	34 (22-108)	75 (6.5-97)	0.662
**Bone marrow blast, %, median (IQR)**	45 (20-70)	50 (23-70)	26 (21-58)	0.334
**Median latency to t-AML, months (IQR)**	4.1 (2.1-13.5)	5.7 (3.0-14.6)	2.9 (1.8-5.6)	0.227
**N. of patients with karyotype abnormalities, n (%)**	17 (73.9)	11 (73.3)	6 (75.0)	0.950

ANC, absolute neutrophil count; t-AML, therapy-related acute myeloid leukemia; IQR, interquartile range.

†*p* values indicate differences between solid tumors and hematologic cancer history cohorts.

**Figure 7:**
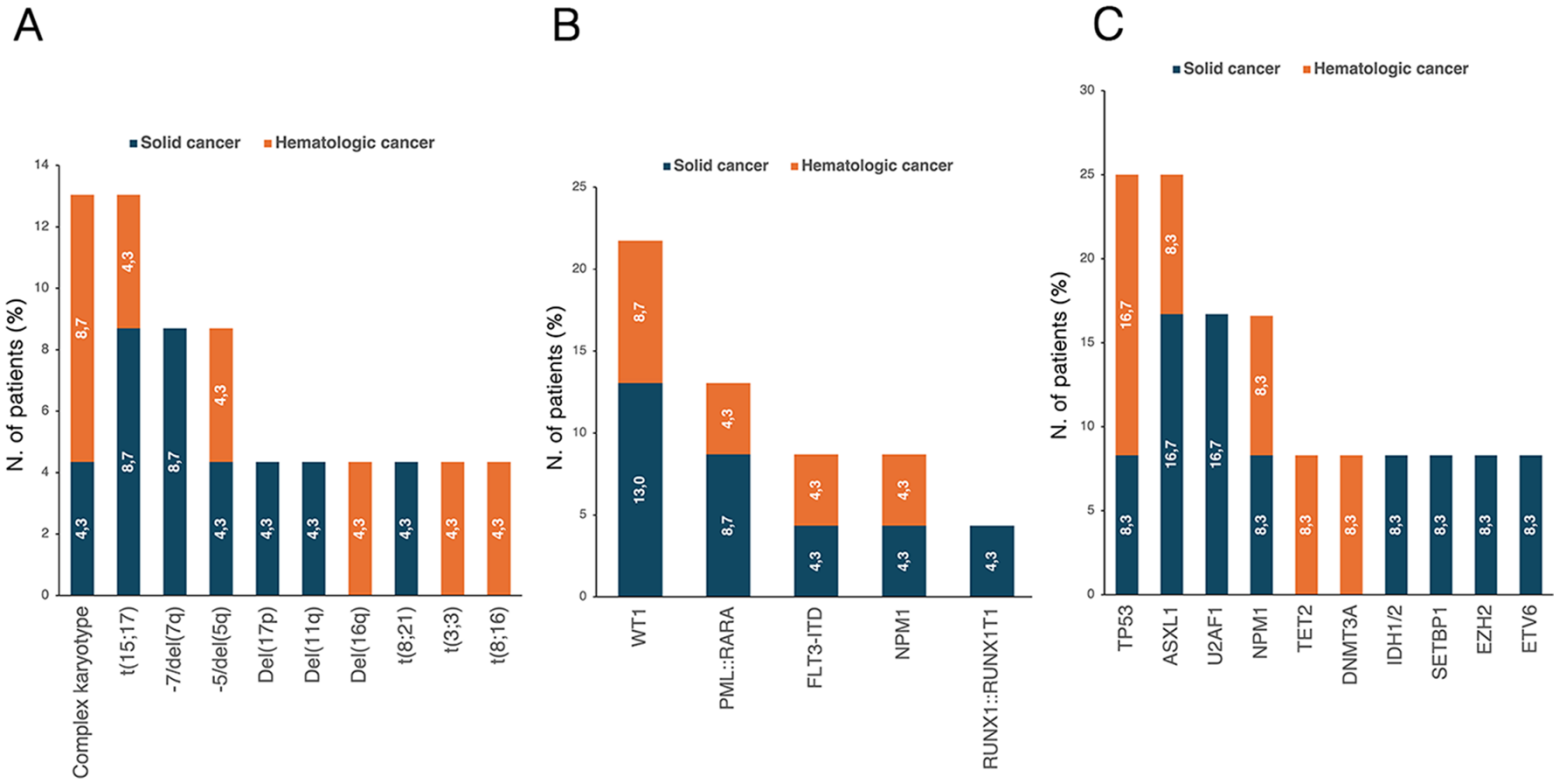
Distribution of **(A)** karyotype abnormalities in evaluable patients (n=23) and molecular mutations detected through **(B)** Real-time PCR (evaluated patients, n=21) and **(C)** NGS (evaluated patients, n=12). The percentages reported within the bars were calculated based on the total number of patients in each respective group. NGS, next-generation sequencing; PCR, polymerase chain reaction.

All patients underwent immunophenotypic analysis at the time of t-AML diagnosis. Specifically, except for the three APL patients, an APL-like phenotype (i.e., CD34^-^ and HLA-DR^-^) was observed in 4 patients (17.4%). Cytogenetic analysis was positive for abnormalities in 17 patients (73.9%). Cytogenetic and molecular abnormalities are pictured in **Figure 7**. Furthermore, 21 patients (91.3%) underwent RT-PCR, which was positive for abnormalities in 11 cases (52.4%). *WT1* abnormalities were the most common (23.8%). Twelve patients (52.2%) underwent NGS testing. The analysis, which tested positive in 9 cases, revealed abnormalities in 9 different genes, mainly involving *TP53* (25.0%), *ASXL1* (25.0%), and *U2AF1* (16.7%).

### 3.7 Treatment and survival analysis in the t-AML cohort

Overall, 16 patients (69.6%) received intensive chemotherapy for t-AML, including CPX-351 in 10 patients (43.5%), standard 3 + 7 regimen in 3 patients (13.0%), 3 + 7+ Gemtuzumab ozogamicin (GO) in 2 patients (8.7%), and one patient received FLAG-IDA regimen. Four patients (17.4%) received non-intensive therapy with venetoclax and azacitidine. APL patients (n=3) received chemo-free therapy according to the APL0406 treatment protocol.

Nine patients (39.1%) were referred for HSCT after a median time of 5.8 months (IQR: 5.1–6.8) from diagnosis. Three patients were at low ELN risk, three at intermediate risk, and other three at adverse risk. Of transplant recipients, 5 patients (55.6%) had received induction therapy with CPX-351, 3 (33.3%) received standard 3 + 7 chemotherapy, and one patient received FLAG-IDA. At a median post-transplant follow-up of 21.8 months (IQR: 14.5–36.3), only one death was recorded due to disease relapse.

**Figure 8:**
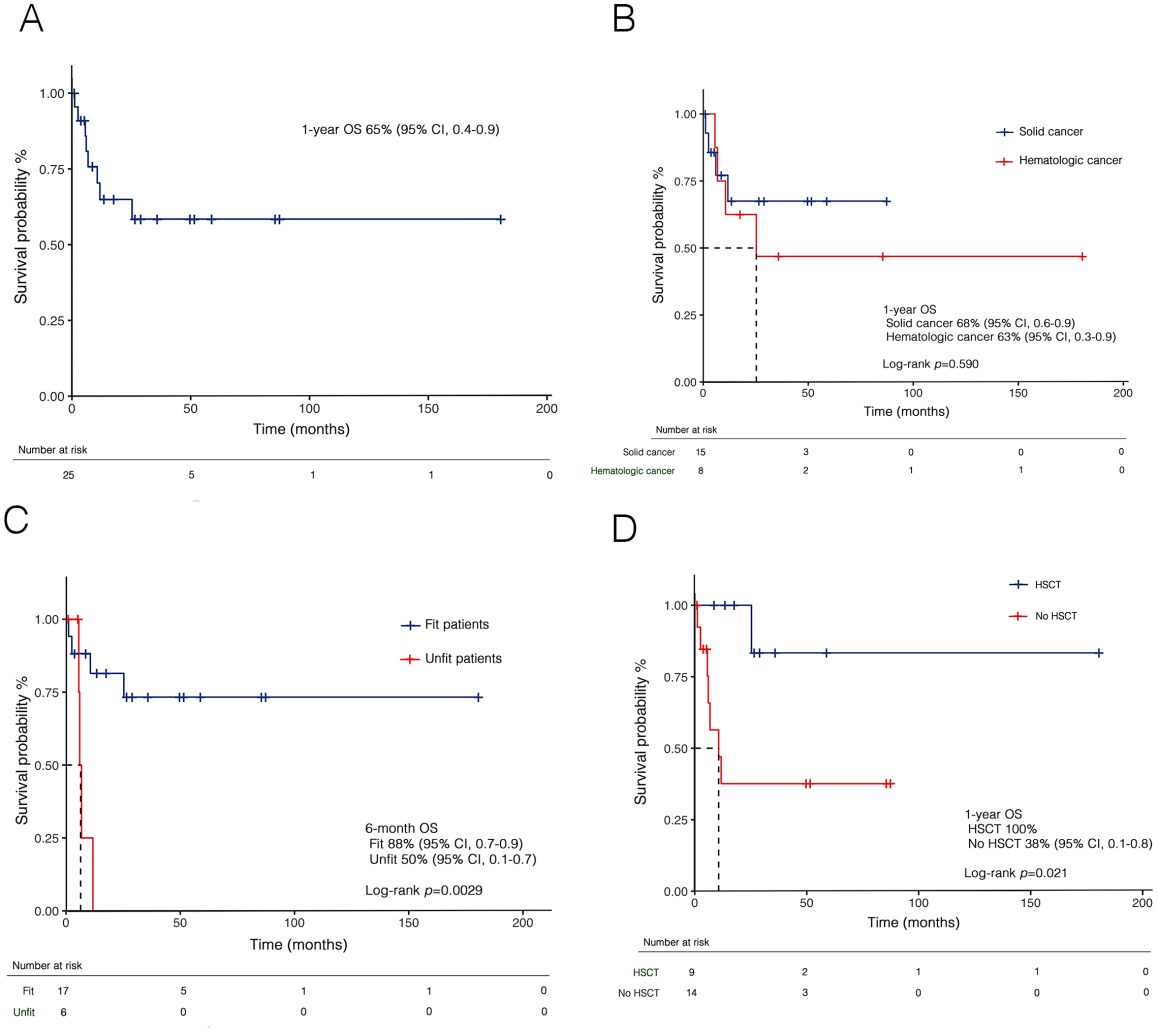
Kaplan-Meier curves for overall survival (OS) in t-AML in the whole cohort **(A)**, and stratified by prior malignancies **(B)**, fitness according to SIE/SIES/GITMO criteria **(C)**, and transplantation **(D)**. HSCT, hematopoietic stem cell transplant; t-AML, therapy-related acute myeloid leukemia; SIE, Società Italiana di Ematologia; SIES, Società Italiana di Ematologia Sperimentale; GITMO, Gruppo Italiano per il Trapianto di Midollo Osseo.

At a median follow-up of 13.5 months (IQR: 5.8–49.2), the 1-year OS of the entire cohort was 65% (95% CI, 0.4–0.9) (**Figure 8A**). No significant differences were observed between the ELN 2022 risk categories [low risk: 100% vs intermediate risk: 75% (95% CI, 0.6–0.8) vs adverse risk: 64% (95% CI, 0.5–0.8), *p*=0.623] or based on primary disease (**Figure 8B**). However, a significant difference emerged based on fitness criteria, with a 6-month OS of 50% (95% CI, 0.1–0.7) for unfit patients compared to 88% (95% CI, 0.7–0.9) for fit patients eligible for intensive therapy (*p*=0.0029) (**Figure 8C**). Moreover, increased survival rates were observed in transplant-recipients compared to non-transplanted patients (*p*=0.021) (**Figure 8D**).

## 4 Discussion

In this retrospective study, we aim to provide new insights into the clinical and prognostic features of t-MN. The demographics and distribution of primary diseases were similar to prior studies (4, 5), with breast cancer being the most frequent malignancy. Indeed, advances in breast cancer diagnosis and treatment have improved survival rates but have also increased the incidence of t-MN (17). Similarly, among hematologic primary diseases, lymphoproliferative neoplasms were the most prevalent, likely reflecting the extensive use of polychemotherapy and the resulting improvements in survival rates (18).

In patients with t-MDS, prior studies have highlighted the superior prognostic accuracy of the IPSS-M compared to the IPSS-R (9, 19). Notably, a higher prevalence of high-risk features is typically observed in t-MDS compared to *de novo* cases (20, 21). However, in our cohort, over 50% of patients were categorized as low to very low risk based on the IPSS-R, consistent with findings from more recent studies (22, 23). Conversely, the molecular score identified a higher proportion of high-risk patients. To our knowledge, this is the first study to specifically analyze the distribution of risk categories between the IPSS-R and IPSS-M in relation to the history of primary malignancy, also demonstrating a slight superiority of the molecular score over the standard one. Patients with prior hematologic cancer exhibited more adverse characteristics, including *TP53* mutation, with a higher proportion classified as high to very high risk by IPSS-M, likely due to greater exposure to polychemotherapy, multiple lines of cytotoxic treatment, and high-risk genetic alterations. Notably, this is highlighted by the higher prevalence of karyotypic abnormalities and a history of hematologic malignancies compared to solid tumors. The additional value of molecular analysis becomes even more pronounced when assessing the risk of progression to AML. In our cohort, the progression rate of 26.5% was consistent with previous reports (20), yet the IPSS-R score surprisingly failed to discriminate this outcome adequately. In contrast, a clear prognostic difference emerged when applying the molecular score. Although not statistically significant, the higher progression rate observed in patients with a history of hematologic neoplasm likely reflects greater biological complexity compared to solid cancer.

An intriguing aspect of our analysis is the observation of an extended latency period in patients harboring *TP53* mutations compared to their *TP53* wild-type counterparts, consistent with recent findings by Hung et al. (24). Similar prolonged latencies have also been reported in other contexts, although not directly compared to wild-type cases (16, 25). At first glance, this observation may appear paradoxical, as *TP53*-mutated clones are generally associated with more rapid progression to advanced, chemo-resistant disease stages (26). However, the leukemogenic mechanisms driven by *TP53* mutations appear to diverge from those of other mutational processes, reinforcing the notion of *TP53*-mutated myeloid neoplasms as a distinct biological entity.

Notably, *TP53* mutations are enriched in clones lacking concurrent DTA mutations, suggesting an alternative leukemogenic trajectory. Indeed, the mutational landscape of t-MN may differ depending on the type of primary malignancy, potentially reflecting distinct selective pressures imposed by previous therapies. *TP53*-mutated clones are well-documented for their resistance to chemotherapy, which predominantly relies on DNA damage-induced apoptosis, and they are recognized as early drivers in the pathogenesis of MDS and AML (27, 28). Moreover, latency duration does not appear to differ significantly between *single-hit* and *multi-hit TP53* mutations (25). In this context, alterations within the bone marrow microenvironment may play a critical role. *TP53*-mutated clones seem to exist at subclonal levels prior to cytotoxic therapy, insufficient to outcompete normal hematopoietic cells or initiate overt leukemia. However, these clones may acquire a selective advantage when therapy-induced microenvironmental changes create conditions favorable for their expansion (29).

The extended latency observed in *TP53*-mutated cases may thus reflect the temporal requirement for therapy-mediated remodeling of the bone marrow niche to establish an environment conducive to the proliferation of therapy-resistant *TP53*-mutated clones. However, the concept of “latency” requires further investigation, particularly in relation to clonal evolution, the role of the bone marrow microenvironment, and whether prolonged exposure to prior cytotoxic therapies acts as a critical trigger in the development of t-MN. It can be hypothesized that the cumulative effect of cytotoxic treatments in prior malignancies resembles the role of reactive oxygen species (ROS) in oxidative stress, where sustained exposure leads to DNA, mitochondrial, and protein damage, ultimately promoting genomic instability. A key limitation of our analysis is the combined evaluation of latency in both t-MDS and t-AML, which may mask the specific contributions of certain mutations to different stages of disease progression.

A crucial aspect of managing t-MN concerns the differing outcomes for t-AML and t-MDS. While survival rates for t-AML have shown modest improvement, likely due to the introduction of CPX-351, similar advancements have not been seen in t-MDS (30, 31). According to the IPSS-R, most t-MDS patients received supportive therapies, with only a small subset treated with HMAs. The high mortality in high-risk t-MDS patients without access to HSCT underscores the lack of effective curative options for this group. In our cohort, HSCT provided a significant prognostic benefit, with markedly improved outcomes in t-AML patients who underwent transplantation. The importance of patient selection was evident, with significant survival differences observed between fit and unfit patients based on the SIE/SIES/GITMO criteria, highlighting their crucial role in treatment decisions. All unfit patients received non-intensive therapy, while fit patients underwent intensive therapy, achieving complete response, which was present in all transplanted patients.

To note, our study has several limitations. First, the limited sample size and retrospective design introduce potential selection and analysis biases. Indeed, molecular data and molecular-based risk stratification were not available for all patients. Furthermore, although not the primary focus of the study, assessing the individual contribution of each chemotherapeutic agent was not feasible due to the widespread use of polychemotherapy regimens and multiple lines of therapy. More importantly, this limitation precluded evaluating how primary disease therapies affect cytogenetic and molecular abnormalities. Nevertheless, our study emphasizes the contribution of molecular analysis to improving the prognostic stratification of patients with t-MDS, particularly in predicting progression to AML, and highlights its critical role in clinical decision-making. Furthermore, there is an urgent need for appropriate therapeutic solutions for patients with t-MDS, for whom HSCT remains the only potential curative option.

## 5 Conclusions

This study provides a comprehensive analysis of the clinical and genetic characteristics of patients with t-MN, highlighting key differences between patients with a history of solid and hematologic tumors and emphasizing the importance of incorporating molecular analysis into the diagnostic and decision-making process. We also underscore the urgent need for effective therapeutic solutions for t-MDS patients, where HSCT remains the only curative option for eligible patients. We also stressed the importance of appropriate patient selection for intensive therapies in t-AML. Overall, these findings reinforce the need for a personalized treatment strategy based on clinical, cytogenetic, and molecular criteria to optimize outcomes in this complex and high-risk population.

## Data Availability

The original contributions presented in the study are included in the article/**Supplementary Material**. Further inquiries can be directed to the corresponding author.
