# Supplementary material for: Impact of primary cancer history and molecular landscape in therapy-related myeloid neoplasms
Source: Front Oncol. 2025 Apr 24;15:1563990. doi: 10.3389/fonc.2025.1563990 (PMC12058663; doi:10.3389/fonc.2025.1563990)
Supplement: Supplementary file 1 [file Table1.docx]

Supplementary Material


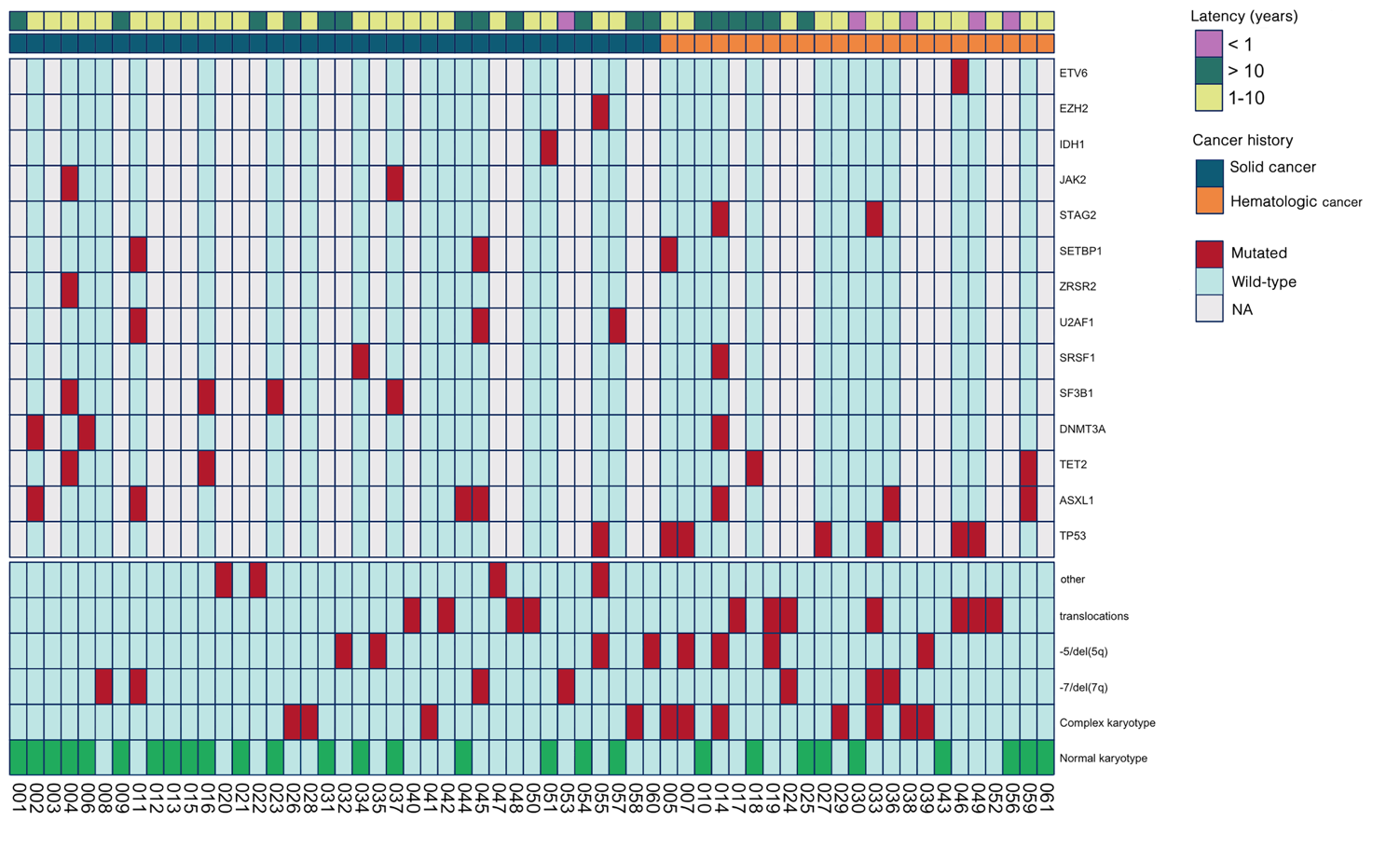


**Supplementary Figure 1.** **Distribution of cytogenetic and molecular abnormalities.** Latency periods were stratified into short (<1 year), intermediate (1–10 years), and long (>10 years). The distributions were further stratified based on a prior history of solid tumors (blue squares) or hematologic malignancies (orange squares). Karyotypes classified as normal are represented in light green. Patient IDs are displayed at the lower axis, while cytogenetic and molecular abnormalities are listed on the right side of the figure.

Abbreviations**:** ID, patient identification; NA, not available
